# AI-generated food stimuli match real counterparts in perceived healthiness and calorie content but not in realism or willingness to eat

**DOI:** 10.1038/s41598-026-66977-1

**Published:** 2026-08-15

**Authors:** Alexander Diel, Leonardo Pimpini, Giuseppe Marrazzo, Finley Sam Mellis, Niels Weber, Tania Josan Lalgi, Laura Bäuerle, Martin Teufel, Alexander Bäuerle

**Affiliations:** 1 Clinic for Psychosomatic Medicine and Psychotherapy, LVR-University Hospital Essen, University of Duisburg-Essen, Essen, Germany; 2 Center for Translational Neuro- and Behavioral Sciences, University of Duisburg-Essen, Essen, Germany; 3 Department of Clinical Psychological Science, Maastricht University, Maastricht, Netherlands

**Keywords:** AI-generated food, AI hyperrealism, genAI, Deepfake, Palatability, Neuroscience, Psychology, Psychology

## Abstract

Visual food stimuli are widely used in food perception and evaluation research, but are often limited by a lack of control of confounding variables. AI-generated food images may be a viable but yet understudied alternative. In a within-subject online experiment, 87 participants rated 60 pairs of AI-generated and pre-validated real visual food stimuli, balanced for calorie content and flavor, on four 100ms visual analogue scales (realism, willingness to eat, calorie content, healthiness). AI-generated visual food stimuli were rated as significantly less realistic and elicited lower willingness to eat compared to real visual food stimuli. Ratings of healthiness and calorie content did not differ between real and AI-generated stimuli. The perceived realism of AI-generated visual food stimuli relative to their real counterparts predicted relative ratings of healthiness and willingness to eat, indicating that AI hyperrealism is associated with higher secondary food-related evaluations. The results show that AI-generated visual food stimuli are perceived similar to real counterparts in aspects of food quality evaluation (healthiness, calorie content), yet not regarding palatability (realism, willingness to eat). Taken together, although AI-generated visual food stimuli can be associated with enhanced food evaluations beyond those of real food, they are perceived as less realistic than their real counterparts.

## Introduction

Food plays an essential role in human life as a main source of nourishment. Visual depictions of food used in research as stimuli to elicit specific responses (here *visual food stimuli*^1^) strongly shape expected palatability, cognitive processing, and appetite responses^2–4^, and are widely used in research as they reliably evoke appetitive, cognitive, and affective responses related to eating behavior^5,6^. Standardized food image databases have been developed with validated food properties and ratings^7–9^; however, food stimulus databases often have limited variability and a small number of available stimuli, especially restricting computational or data-driven approaches for which large datasets are needed^10^. Even the largest open stimulus sets remain modest in size (e.g., food-pics with 568 images). This constrains controlled variability in stimulus selection, e.g., when the goal is to systematically manipulate one target attribute (e.g., healthiness) while controlling others (e.g., flavor, portion, lighting), as it will quickly exhaust the available matched stimuli. For example, to fill all cells for food stimuli varying across three 10-point attribute scales, 1,000 pre-validated food stimuli would be necessary; hence, current visual food stimulus databases do not provide sufficient variability for research questions that need such controlled stimulus sets.

Recent advances in generative artificial intelligence (AI) led to the development of AI-based image generators with the capacity to create deceptively realistic synthetic content^11^. AI-generated visual food stimuli, for example, have already found use in marketing and advertising, and are largely present on social media^12,13^. Given the relative ease and flexibility of the generation of AI visual food stimuli, their use may provide an efficiency advantage.

In the context of clinical research, AI-generated visual food stimuli may be used as alternatives to real counterparts: Generative AI can easily generate visual food stimuli with varying degrees on relevant scales, such as naturalness, healthiness, portion, texture, plating style, etc., while keeping other variables constant. Such controlled variety is difficult to achieve with real food stimuli sourced from the web (e.g., Google images, Stockphotos), as such images cannot be manipulated orthogonally along a target dimension, and they often carry uncontrolled confounds in lighting, viewing angle, background, resolution, and branding that are known to vary across food-image sources and to be left unspecified in prior work^7,10^.

However, transferability between real and AI-generated visual food stimuli is not guaranteed: AI-generated food labeled as such is perceived as less appealing^14^, imperfections in AI-generated food stimuli may appear eerie^15^, and VR simulation of certain food-related behaviors (e.g., eating) cannot reproduce real eating behavior^16^. Hence, the transferability between real and virtual or AI-generated food ought to be investigated to validate their usability for research.

Research on the evaluation of AI-generated visual food stimuli, especially compared to real counterparts, remains sparse and limited in terms of methodology, rating, and variety of depicted food dishes^14–18^. Across currently present work, AI-generated visual food stimuli tend to show lower evaluations: Stright et al.^17^ found that participants reliably detected AI-generated visual food stimuli and that perceived AI origin was negatively associated with appeal, naturalness, perceived healthiness, and willingness to eat. Califano & Spence^14^ found that AI-generated food was preferred over real food only when their synthetic nature was not disclosed. Such effects are further modulated by stimulus- and person-level factors: more processed food is more likely to be correctly recognized as AI-generated^14^ while errors in AI-generated food are perceived as eerie^15^. Furthermore, food neophobia (a trait associated with the dislike of new types of food) was associated with eeriness of AI-generated food^15^, while younger individuals appear more susceptible to AI-generated food^18^. However, existing studies remain limited for the present purposes: The stimulus sets have been small and/or unbalanced (e.g., five food types not matched on calorie content or flavor^17^, or not properly paired for real and AI-generated counterparts^15^. Furthermore, no study has yet compared a broad range of matched, pre-validated real and AI-generated food stimuli across multiple evaluative dimensions.

Four stimulus evaluation dimensions are selected here as dependent variables based on the use cases of visual food stimuli: realism, healthiness, willingness to eat, and calorie content. These dimensions are selected to encompass food as appetitive stimuli (e.g., use for cue-reactivity, marketing, or exposure paradigms) and for the subjective perception of food properties and representation (e.g., relevant for dietary, menu, and clinical research). Hence, the scales ought to measure the comparability of AI-generated and real food stimuli in terms of perceptual fidelity (*realism*), appetitive affect (*willingness to eat*), and inference of nutritional values (*healthiness*,* calorie content*).

*Realism* is here defined as perceived authenticity of the stimulus’ origin, i.e., the degree to which an image is judged to depict a genuine photograph or real food, consistent with research on the perception of AI-generated faces (e.g., *photorealistic*)^19,20^. Prior work found AI food to be less realistic than real food^18^, particularly in highly processed food^14^. Realism is related to but distinct from photographic quality (conflated by features related to the image rather than food quality), naturalness (which may conflate with the food’s level of processing), attractiveness or aesthetic appeal (which may conflate with food presentation or other factors), and familiarity (conflated by a viewer’s personal experience with the food depicted).

*Willingness to eat* ratings indicate the appetitive affect and approach-motivational response most relevant to appetitive and applied uses of visual food stimuli: It allows an assessment of whether AI-generated food stimuli elicit eating intentions comparable to real food. Perceiving food as AI-generated has been associated with a reduced willingness to eat^17^ and with lower preference once AI origin is disclosed^14^.

*Healthiness* and *calorie content* ratings relate to cognitive inferences of the nutritional properties of a food. Such ratings can indicate whether AI-generated visual food stimuli may substitute real counterparts when subjective estimation of food properties is relevant. Healthiness ratings have been lower for AI-generated food stimuli^17^ and may be influenced by the detection of small errors in AI-generated visual food stimuli indicating potential health hazards: Disgust is an emotional reaction of food rejection that evolved as part of a disease-avoidance system, triggered by sensory cues such as abnormal appearance or signs of decay or spoilage which may threaten health if consumed^15,21^. AI-generated images may contain subtle imperfections and structural anomalies which can trigger disease-avoidance reactions and decreased healthiness ratings. Calorie content ratings, by contrast, have not been investigated in any prior AI-real comparison of visual food stimuli. As an estimator of a food attribute, comparisons of calorie content ratings test the suitability of AI stimuli for dietary, menu, and clinical use.

Research on AI-generated visual food stimuli shows that negative evaluation of AI-generated food is associated with perceiving the visual food stimuli as AI-generated^14–18^. It could be that the lack of perceived realism drives negative evaluations. Meanwhile, research on face processing using AI-generated faces recently proposed an AI hyperrealism effect, showing that AI-generated faces are perceived as more real, attractive, and trustworthy compared to real faces^19^. Hyperrealistic stimuli may act similarly to superstimuli, i.e. exaggerated stimuli that elicit stronger stimulus-related reactions, due to their hyperreal appearance^22,23^, driven by an AI’s tendency to generate prototypical or best-fitting exemplars. When applied to visual food stimuli, stimuli that appear hyperreal (i.e., more realistic than real food) are expected to elicit higher food-related evaluations (e.g., healthiness, willingness to eat) compared to real counterparts.

### Research questions and hypotheses

In total, the hypotheses are as follows: (1) AI-generated visual food stimuli are rated significantly lower in realism, healthiness, and willingness to eat, and differ in calorie content compared to real counterparts; (2) Differences in realism ratings of AI-generated visual food stimuli predict differences in ratings of calorie content, healthiness, and willingness to eat.

**Fig. 1:**
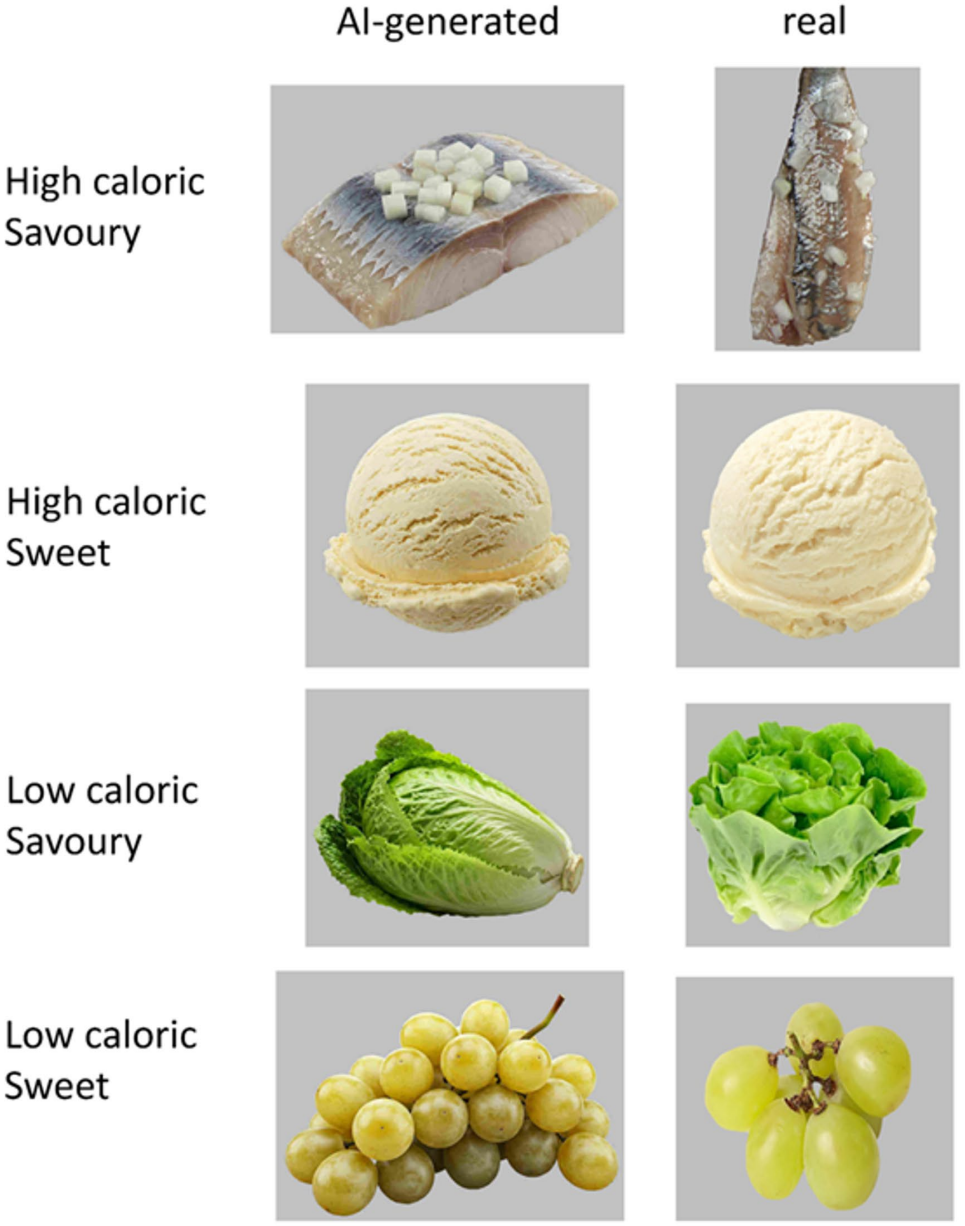
Food stimuli across conditions. Example visual food stimuli (AI-generated and real) for each category: herring with onions (high caloric savory), vanilla ice cream (high caloric sweet), lettuce (low caloric savory), grapes (low caloric sweet).

In addition, exploratory analyses are conducted with self-reported hunger, food neophobia, and BMI as moderators (Fig. 1).

## Results

**Fig. 2:**
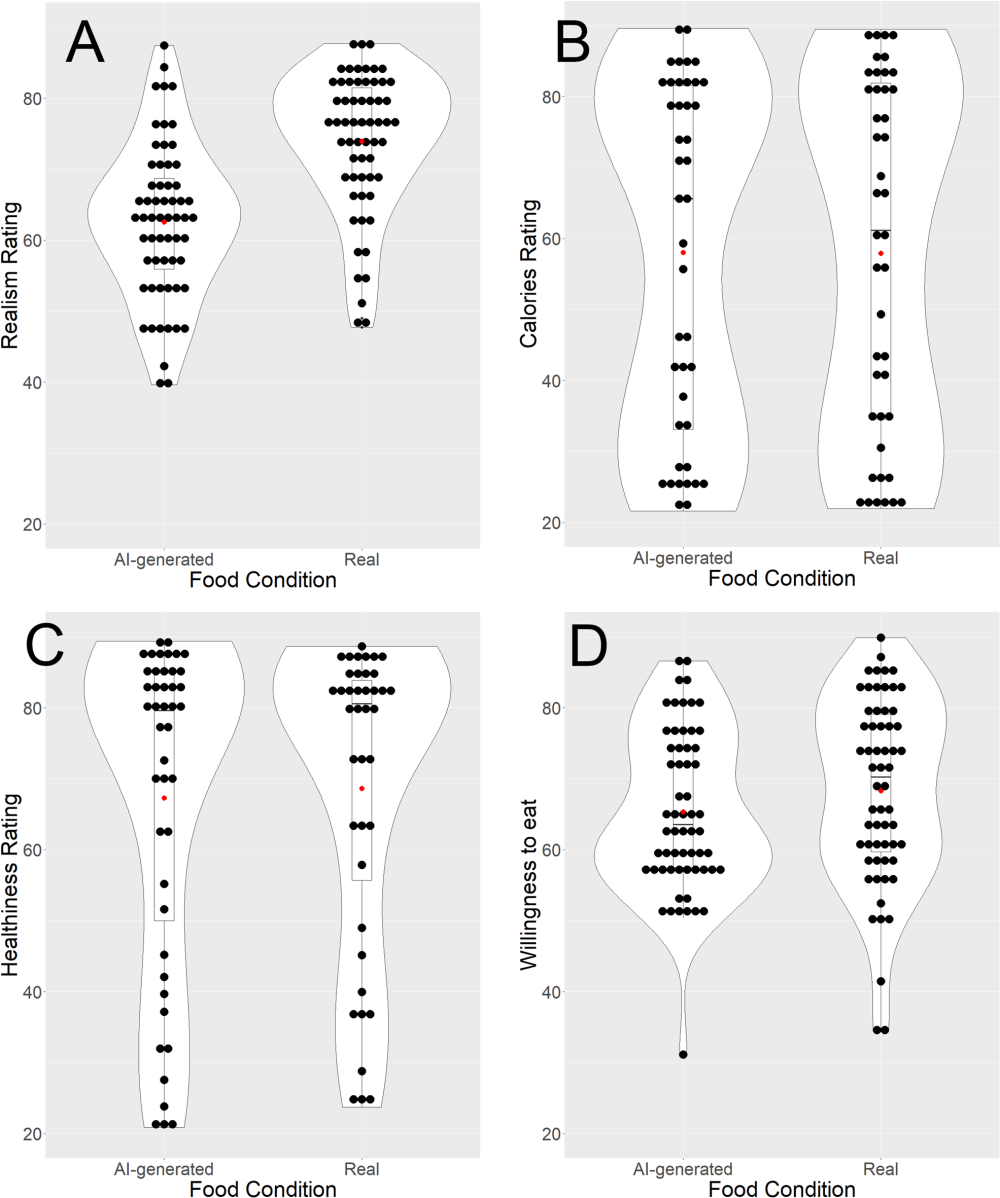
Food ratings across conditions and scales. Violin plots and boxplots with average realism (**A**), calorie content (**B**), healthiness (**C**), and willingness to eat (**D**) ratings of AI-generated and real food. The red dot signifies the mean and black dots indicate averaged food stimuli.

Results show that real visual food stimuli were rated as significantly more realistic (*F*(1,117) = 23.22, *p* < .001, *R*^2^_c_ = 0.50, β = 11.14) and elicited higher willingness to eat (*F*(1,59) = 11.97, *p* = .001, *R*^2^_c_ = 0.30, β = 3.06) compared to AI-generated counterparts, while no differences were observed for ratings of calorie content (*F*(1,62) = 4.18, *p* = .045, *R*^2^_c_ = 0.83, β = -0.70) or healthiness (*F*(1,59) = 0.04, *p* = .834, *R*^2^_c_ = 0.86, β = -0.08) under the Bonferroni-corrected threshold. Control variables (BMI, food neophobia, hunger) did not predict any of the outcomes after Bonferroni correction; statistics are summarized in Table A3. Significant and non-significant results remained when outliers were included in the analysis (Table A1). Full statistics including estimated marginal means, standard errors, and confidence intervals are reported in Table A2. Results are summarized in Fig. 2.

Post-hoc analyses adding calorie content (low vs. high) and flavor (savory vs. sweet) as interaction variables with food condition (AI-generated vs. real) show that the significant effect of condition on realism remained (*F*(1,114) = 23.03, *p* < .001, *R*^2^_c_ = 0.51, β = 12.40), with no other effect significant (calorie content: *F*(1,58) = 0.97, *p* = .329, β = -3.79; flavor: *F*(1,58) = 0.38, *p* = .538, β = 0.66). For estimated calories, both calorie content (*F*(1,59) = 246.30, *p* < .001, β = 51.87) and flavor (*F*(1,59) = 14.43, *p* < .001, β = 12.36) predicted ratings (*R*^2^_c_ = 0.83) while the effect of food condition remained not significant (*F*(1,60) = 4.10, *p* = .047, β = -1.06); the same pattern was observed for healthiness (*R*^2^_c_ = 0.86; calorie content: *F*(1,59) = 148.38, *p* < .001, β = -51.81; flavor: *F*(1,59) = 8.67, *p* = .005, β = 12.46; condition: *F*(1,57) = 0.04, *p* = .843, β = -0.46. Furthermore, calorie content predicted willingness to eat (*F*(1,58) = 11.89, *p* = .001, β = -9.49) and food condition (*F*(1,59) = 11.01, *p* = .002, β = 2.33) remained significant (*R*^2^_c_ = 0.30). No interaction between food type (calorie content, flavor) and food condition (AI-generated vs. real) was found for any measures, indicating that the observed effects of food condition remain stable across different food varieties. Further post-hoc analyses with normative calorie content predicting calorie ratings (*R*^2^_c_ = 0.83) showed that normative calorie content significantly predicted calorie ratings (*F*(1,59) = 169.20, *p* < .001, β = 0.16) while condition remained not significant (*F*(1,64) = 2.66, *p* = .108, β = -0.88), and the interaction between normative calorie content and condition was also not significant (*F*(1,61) = 0.09, *p* = .767, β < 0.001). In an additional post-hoc analysis, residual variability of calorie ratings did not differ between AI-generated and real stimuli (residual SD ratio = 0.995, likelihood-ratio test χ^2^(1) = 0.10, *p* = .75), indicating no differences in calorie rating precision between AI-generated and real food stimuli.

A further post-hoc analysis on learning effects occurring throughout the experiment (*R*^2^_c_ = 0.50) showed that while stimulus condition significantly predicted realism ratings (*F*(1,182) = 16.26, *p* < .001, β = 12.94), there was no significant effect of trial number (*F*(1,519) = 3.02, *p* = .083, β = 0.001) nor a significant interaction between condition and trial number (*F*(1,155) = 0.74, *p* = .391, β < 0.001). Another post-hoc analysis on the effects of stimulus counterpart proximity (*R*^2^_c_ = 0.50) revealed a significant main effect of food condition on realism (*F*(1,134) = 20.93, *p* < .001, β = 10.96) but no significant main effect of counterpart proximity (*F*(1,7366) = 1.64, *p* = .20, β = 0.01), or an interaction between these two (*F*(1,7424) < 0.001, *p* = .983, β < 0.001). For willingness to eat (*R*^2^_c_ = 0.32), only the main effect of proximity was significant (proximity: *F*(1,7417) = 6.46, *p* = .011, β = 0.03; condition: *F*(1,227) = 2.54, *p* = .112, β = 1.81; proximity-condition interaction: *F*(1,7158) = 0.09, *p* = .766, β = 0.01).

Finally, an exploratory post-hoc analysis on sex effects on realism ratings (Table A4) showed that the observed realism differences between real and AI-generated stimuli were larger for male (difference = 18.66, SE = 3.08) compared to female participants (difference = 7.69, SE = 2.42).

### AI hyperrealism effects

As AI-generated visual food stimuli were rated as less realistic than real counterparts, the main AI hyperrealism effect was not found. Moderator analysis results show that the differences in realism ratings significantly predicted differences in healthiness (*F*(1,46) = 18.39, *p* < .001, *R*^2^_c_ = 0.08, β = 0.04), and willingness to eat (*F*(1,91) = 58.43, *p* < .001, *R*^2^_c_ = 0.21, β = 0.20) but not calorie content (*F*(1,51) = 1.13, *p* = .293, *R*^2^_c_ = 0.03, β = -0.01). Exploratory secondary analyses furthermore showed that realism ratings predicted healthiness (*F*(1,100) = 47.35, *p* < .001, *R*^2^_c_ = 0.72, β = 0.04) and willingness to eat (*F*(1,46) = 36.47, *p* < .001, *R*^2^_c_ = 0.62, β = 0.27) across both real and AI-generated visual food stimuli, but not estimated calories (*F*(1,36) = 0.03, *p* = .862, *R*^2^_c_ = 0.62, β = 0.01).

**Fig. 3:**
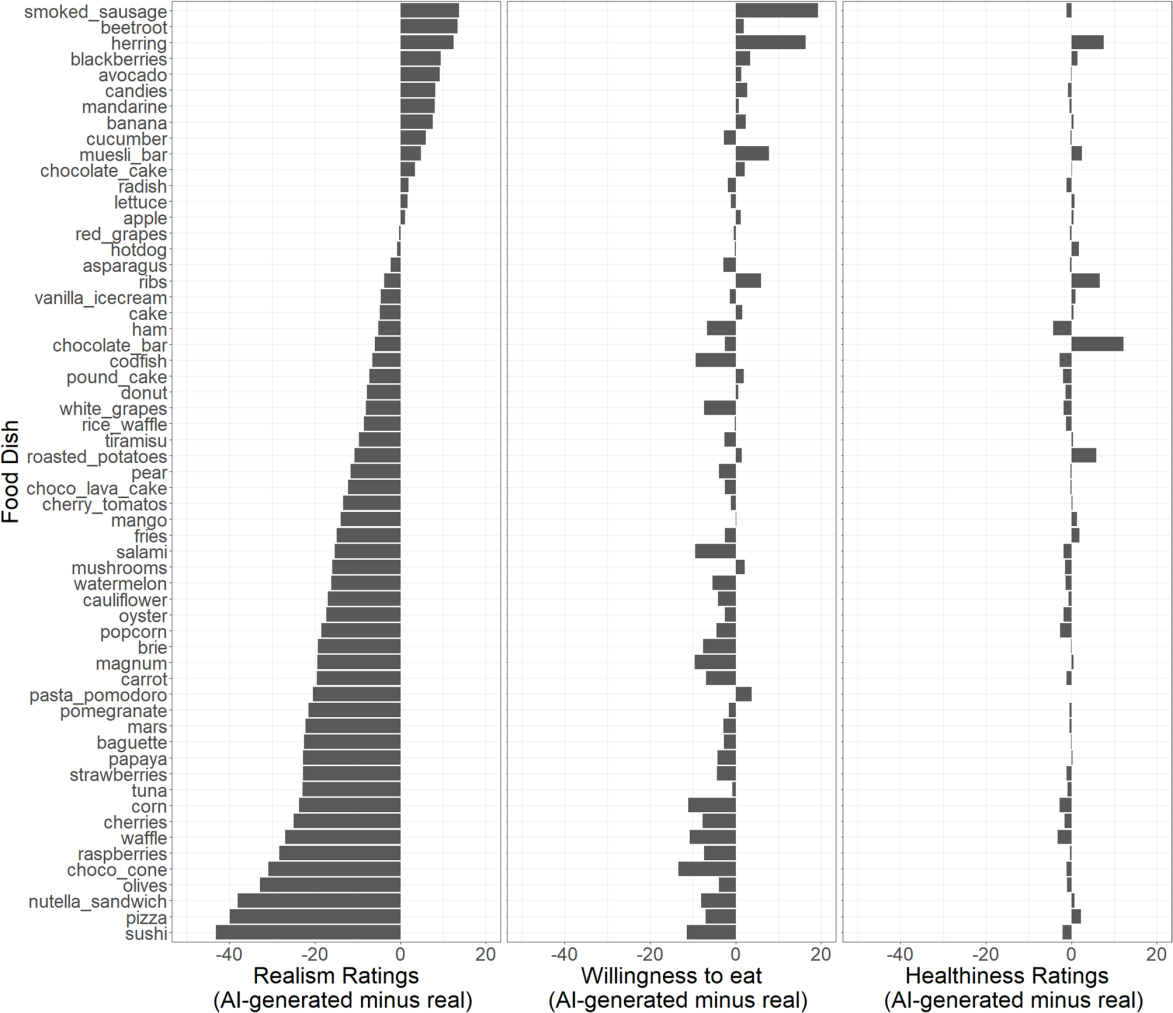
Relative evaluation ratings. Relative (AI-generated minus real counterparts) ratings of realism, willingness to eat, and healthiness of food stimuli sorted by relative realism ratings.

Finally, Fig. 3 shows that AI-generated food stimuli that were more realistic than real counterparts also tended to be rated with higher relative willingness to eat: out of the 14 stimuli with higher realism ratings for AI-generated compared to real counterparts, 11 also showed higher willingness to eat ratings for AI-generated compared to real counterparts. However, this pattern is less clear for healthiness ratings.

Hence, hypothesis 2 is partially supported, especially in that the subset of hyperrealistic food stimuli (operationalized by higher AI-generated realism ratings compared to real counterparts) also tended to show higher relative ratings in willingness to eat.

## Discussion

AI-generated visual food stimuli find increasing uses in various contexts, especially in the digital environment such as social media, and have the potential to be used as alternative to real food stimuli in clinical research on appetite and eating disorders. The goal of the present study was to investigate the perception and evaluation of AI-generated compared to real visual food stimuli. It was found that AI-generated visual food stimuli are rated as less realistic and participants were less willing to eat those compared to real counterparts. Meanwhile, healthiness and calorie content did not differ between conditions. Furthermore, differences in realism ratings between AI-generated and real visual food stimuli significantly predicted differences in healthiness and willingness to eat ratings, indicating that the more (or less) realistic an AI-generated visual food stimulus compared to real counterparts, the more (or less) healthy and appealing it appears. This can be seen in Fig. 3: 14 AI-generated visual food stimuli were hyperrealistic (i.e., higher realism ratings compared to real counterparts), and 11 out of those also showed relatively higher willingness to eat ratings. Meanwhile, out of 46 AI-generated stimuli not rated as more realistic than their real counterparts, 40 were also rated with lower relative willingness to eat.

These results are consistent with previous research showing that AI-generated visual food stimuli are perceived as less realistic than real counterparts^17^, and that the perception of AI-generated visual food stimuli as unrealistic (or AI-generated) is associated with negative evaluations^14,17^. Given the effect of label manipulation of food ratings^14^, it seems that perceiving AI-generated visual food stimuli as AI-generated lowers food evaluation, rather than the other way around.

The present findings show that a general AI hyperrealism effect observed for AI-generated faces^19^ cannot be replicated for AI food stimuli in this study. Instead, AI-generated visual food stimuli were on average perceived as less realistic compared to real food variants, despite pre-selection and stimulus curation procedures. Only a subset (14 of 60 pairs) could be considered hyperrealistic. Face hyperrealism has been attributed to generative models producing prototypical faces near the center of the face space^19^, where averageness drives perceptions of prototypicality, realism, and attractiveness. A food analogue to the face space has, however, never been established, with no evidence that food has an analogous dimensional prototype structure to the face space. Hence, the results suggest that general AI hyperrealism effects are not domain-general, and may depend on domain-specific processing mechanisms (e.g., face space and prototypicality for faces).

However, the present findings also suggest that AI hyperrealism in a subset of visual food stimuli was associated with enhanced food evaluations, especially appetitive ones. This association is consistent with a superstimulus account, under which exaggerated stimuli elicit enhanced stimulus-related responses. However, the present results cannot test this causal account as realism was not manipulated. Hyperrealistic AI-generated visual food stimuli may accordingly be of interest where the goal is to exaggerate food-related perceptions, such as in clinical research or advertisement. In that case, however, a pre-evaluation of food realism would be necessary to select appropriate hyperrealistic visual food stimuli.

While real and AI-generated visual food stimuli did not differ in healthiness ratings, differences in realism ratings between matched pairs predicted their differences in healthiness ratings. The condition effect concerns the mean difference across all pairs while the item-level analysis concerns covariation among matched pairs. The two results are also quantitively consistent in that the item-level slope of β = 0.04 healthiness points per realism point and mean difference of 11.14 realism points indicate a mean healthiness difference of 0.45 points on a 100-mm VAS, which lies within the observed confidence interval for the condition effect. The results indicate that relative realism tracks relative perceived health among matched pairs while differences in healthiness ratings between conditions are negligible.

Interestingly, calorie content ratings did not significantly differ between AI-generated and real visual food stimuli, and neither did realism ratings predict calorie content. It is possible that calorie content, as an information-based evaluation based on inferential, category-based judgments according to the food’s identity^24^, may be less sensitive to the surface perception of visual food stimuli as real or AI. In this context, condition (real vs. AI-generated) did not predict calorie content estimation while calorie content (low vs. high caloric) did, indicating such inferential, category-based reasoning based on the food identity which remains constant across real and AI-generated pictures^24^. Meanwhile, AI generation would alter the perceptual surface qualities of an image while preserving the depicted food’s identity and category, which would not influence inferential judgments based on the food category and prior knowledge. Future research can investigate perception-based versus category-based judgments of AI-generated food. Hence, AI-generated visual food stimuli may find use as alternatives to real counterparts in instances when properties of food (e.g., calorie content) are to be subjectively estimated.

Alternatively, a lack of difference between the calorie estimations of AI-generated and real visual food stimuli may be due to humans’ general difficulties in calorie estimation^25,26^ which may reflect as a low accuracy and high variation for calorie content ratings for both AI-generated and real stimuli. However, calorie content was significantly different between low-caloric and high-caloric food, indicating that ratings can discriminate real caloric differences, supporting criterion validity of the scale. Furthermore, the residual variability of calorie ratings did not differ between AI-generated and real images, indicating that AI-generated images did not yield noisier calorie estimates. Finally, normative calorie ratings of the presented food dishes could predict calorie ratings regardless of condition. Hence, the null effect in calorie rating differences between conditions is unlikely to be explained by general difficulty estimating calories.

The present findings have implications for clinical research on eating disorders (ED) using visual food stimuli and food-cue paradigms. EDs are characterized by altered responses to food-related cues and cognitive and affective appraisals^5,27–29^. Hence, uncontrolled properties of the stimuli may affect approach-avoidance responses specific to EDs and thus risk compromising task validity. Generative AI can be used to generate images of food with controlled variations in a variety of variables, such as calorie content, flavor, or portion size. This would, for example, allow the generation of a series of standardized stimuli with a controlled increase of portion size (e.g., by 10% steps). While AI-generated visual food stimuli in this study elicited lower appetitive responses compared to real counterparts on average, individual hyperrealistic stimuli may be able to amplify food evaluations beyond those measured with real visual food stimuli. For ED research and intervention, hyperrealistic stimuli may be useful for the creation of large, customizable stimulus sets; however, pre-validation would be necessary.

Food neophobia, BMI, and acute hunger did not affect the main results. However, given the low proportion of highly neophobic indivuduals, as is typical of unselected samples, the results do not exclude potential effects that emerge only at extremes. Analogous arguments can be made for acute hunger levels and BMI. Future research may investigate such moderator effects with a higher rate of extreme values for food neophobia, hunger, and BMI.

Furthermore, AI-generated visual food stimuli can be easily personalized, for example for “trigger food” that is specific to individual patients. In addition, synthetic representations of food already find use in clinical research: virtual reality (VR)-based exposure therapy to food stimuli is used in the assessment and treatment of ED symptoms^30^, and may even outperform contemporary therapy in otherwise treatment-resistant cases^31^.

Taken together, these findings highlight practical implications for content and environments in which food choices are shaped by visual representations. For instance, in food-delivery apps or online grocery stores, slightly enhanced or hyperrealistic AI-generated depictions of meals could unintentionally increase consumers’ perceptions of healthiness or desirability, even when the actual nutritional profile of the food does not justify such impressions. Consequently, AI-driven image optimization algorithms — commonly used to refine lighting, texture, or color saturation — might influence consumer behaviors by shifting how ‘real’ or appetizing the food appears. Nevertheless, given that AI-generated visual food stimuli were on average less realistic than real counterparts, reliance on fully automatic enhancers may backfire without proper evaluation of food realism.

Furthermore, these results raise questions about the ethical deployment of AI-generated visual food stimuli in advertising, public health messaging, and menu design. While restaurants or food manufacturers may rely on hyperrealistic AI-generated images to make dishes appear more appealing, public health campaigns could intentionally use hyperrealistic portrayals of healthy foods, such as vegetables or low-calorie meals, to enhance positive evaluations and increase willingness to choose healthier options. However, this also underscores the need for transparency: if consumers learn that appealing food imagery is AI-generated, and if such recognition reduces perceived realism, the persuasive impact of the image may rapidly diminish.

Therefore, future research could investigate how practices, platform context, and viewer expectations interact with AI hyperrealism to shape food-related assessment and behaviors.

Given the within-subject design, effects caused by the exposure of real or AI-generated counterparts could have influenced the results, e.g., via learning effects. However, no interaction between condition and trial number was found in this study, showing no evidence of a learning effect. Prior research on the detection performance of real and synthetic stimuli shows no learning effects across trials unless feedback is provided^20,32,33^; as no feedback was provided in this study, participants had no objective categorization information available to refine detection strategies across trials. Similarly, perceiving counterparts in a row could elicit carry-over effects; however, the post-hoc analysis did not reveal any significant proximity-condition interaction effects on realism or willingness to eat ratings; hence, no evidence was found for carry-over effects caused by perceiving real and AI-generated counterparts after each other.

Rating scales were presented in a fixed order. Carry-over effects from the realism rating onto subsequent evaluations (healthiness, willingness to eat) cannot be excluded. Randomizing rating order or counterbalancing rating scales is recommended for future research.

Another limitation concerns the origin of the observed condition effects: Participants did not assess the binary classification of AI-generated vs. real stimuli. Bottom-up effects driven by the images’ perceptual realism qualities cannot be dissociated from top-down effects driven by participants’ expectations upon judging a stimulus as AI-generated or real. However, stimuli were presented without labels, hence categorizations and emerging top-down effects were not induced experimentally, beyond an initial set-level expectation that AI-generated stimuli are present. Hence, categorization and top-down effects must have emerged from the initial bottom-up assessment of the stimuli, entangling perception- and expectation-based effects. Nevertheless, a definitive dissociation requires the manipulation of condition and label, which can be investigated in future work.

Food dishes vary in familiarity, which could influence perceived realism, e.g., via the detection of noise or artifacts due to a higher level of perceptual familiarity with the food dish. Although food stimuli included were selected by recognizability in the pilot study (food stimuli with less than 66.67% recognizability were excluded), effects may nevertheless occur within the familiarity variation of more familiar dishes. Although condition interactions with food type (low/high caloric, savory/sweet flavor) were not significant, indicating stability of effects across the stimulus set, familiarity was not directly measured. Future research may investigate whether food familiarity moderates the effect of food condition on perceived realism.

The stimuli used in this study were standardized with monotonous gray background to isolate the observed effects of the food depiction itself. This standardization further allows direct comparability to standardized food stimuli used in research where gray or white backgrounds are common^7,8,34^. Meanwhile, food imagery encountered in practice (e.g., social media, restaurant menus, delivery platforms, advertising) often appears in richer visual contexts that may co-vary or interact with the image source: For example, a styled, natural environment (food on a table in a restaurant with a warm, dim candle light) may raise perceived authenticity and desirability. While the current findings focus on the perception of AI-generated versus real food under controlled conditions, they may not be generalizable to naturalistic presentations. Further research may replicate these results using images of food in more varying, natural visual environments.

Realism was operationalized with a single scale. Despite being conceptually different from related concepts such as naturalness or attractiveness, a single-item measure may not reliably isolate these correlated dimensions. Although a single scale of realism may capture a measure holistic impression of realism, future work may use separate scales to test their independent contributions.

The sample contained more male compared to female participants (57 vs. 23), which may confound the effects. An exploratory post-hoc analysis on sex effects on realism ratings (Table A4) shows a significant sex effect and sex-condition interaction: the observed realism difference between food condition is stronger in male compared to female participants. Due to imbalanced sex distribution, the observed realism effect may be inflated by the higher number of male participants. Future research may aim to replicate results in a sex-balanced sample, and further investigate sex effects on the perception of AI-generated food.

Because realism was measured via a continuous scale without binary categorization (real/AI-generated), it is unclear to what degree uncontrolled covariates of realism may have influenced food evaluations like willingness to eat. A categorical recognition measure in addition to a continuous scale, or a design crossing condition with label, would more directly isolate the contribution of categorization as real/AI-generated, which we recommend for future work.

As the goal was to investigate AI-generated visual food stimuli as potential alternatives to real counterparts in the context of potential use cases (e.g., research material, advertising), two curation procedures (prompt refinement with pre-selection and pilot study validation) were used to select AI stimuli. As a consequence, the included AI-generated stimuli represent relatively error-free variants. However, this process introduces a selection bias towards more realistic AI-generated stimuli, and the observed realism difference is likely an underestimate for unfiltered stimulus selection. Prior AI food studies^14,15,17,18^ did not report any selection procedures (or lack thereof), so their potential impact cannot be compared against existing work. Consequently, the present findings generalize to curated AI-generated visual food stimuli but not sufficiently to unfiltered ones.

## Conclusion

The use of AI-generated content becomes increasingly widespread: AI-generated visual food stimuli are used in advertisement, menu plans, and marketing research. However, the perception and evaluation of AI-generated visual food stimuli remains understudied, especially across a variety of measures and food dishes. We find that across food qualities (caloric content, flavor), AI-generated visual food stimuli are perceived as less realistic and less appealing than real counterparts, yet no difference was found for ratings of calorie content or healthiness. Furthermore, hyperrealism in AI-generated visual food stimuli was associated with higher health ratings and willingness to eat. The results suggest that AI-generated visual food stimuli are rated similarly to real counterparts for estimations of properties the dish (e.g., calorie content) but not subjective ratings (e.g., willingness to eat), and that hyperreal AI-generated food are associated with enhanced food-specific ratings.

## Methods

The study consists of an online within-subject experiment on the evaluation of real and AI-generated visual food stimuli. The experiment was preregistered at osf.io/drb3s. A within-subject design was used to allow appropriate comparison of real and AI-generated visual food stimulus counterparts by comparing matched ratings within participants. Meanwhile, a between-subject design would confound condition and participant effects which would not allow direct comparisons between ratings of AI-generated and real food counterparts.

### Participants

Power analysis was conducted using PANGEA^35^. A default small effect size was chosen as a smallest effect of interest to test for the presence of small yet significant differences between conditions. Within-subject power analysis, an alpha of 0.05, a small effect size of *d* = 0.25 and a power of 1-β = 0.8 revealed that *n* = 87 participants were sufficient. Participants were recruited online via Prolific, an online participant recruitment platform known for quality data^36^, and included if they possessed sufficient German language skill to partake in the experiment. The website is designed so that only viable participants could view the study. A total of *n* = 87 complete datasets were recruited. Three participants were excluded for completion times below 16 min (half the sample median) and replaced through re-recruitment to meet the preregistered target of *n* = 87. Individuals with diets restricting consumption of food included in the stimuli (e.g., vegetarianism, veganism) were excluded via Prolific filters. The website Prolific requires participants to verify their identity via ID, prevents duplication via IP validation, and enrols participants in active fraud monitoring; hence, the platform’s layered protection minimizes risk of bot accounts or responses which may contaminate research data. Participant demographics are summarized in Table 1.

**Table 1:** Descriptive data on participant gender, age, BMI, acute hunger, and Food Neophobia scores. Food Neophobia scores were reverse-transformed from 100-mm VAS to into 7-point scales. Highly neophobic and neophilic individuals were identified using the 1 SD criterion^37,38^.

Demographic	Value
Total number	*N*_total_ = 87
Gender	*N*_male_ = 57 *N*_female_ = 23 *N*_other_ = 1 Not reported = 6
Age	*M*_age_ = 36.74 *SD*_age_ = 11.54 range_age_ = [19, 65]
BMI	*M*_BMI_ = 25.53 *SD*_BMI_ = 5.53 range_BMI_ = [18.12, 61.73]
Hunger level (0-100)	*M*_hunger_ = 28.96 *SD*_hunger_ = 26.76 range_hunger_ = [0, 97]
Food Neophobia (reverse-transformed)	*M*_FN_ = 27.78 *SD*_FN_ = 9.14 Range_FN_ = [11, 61] Highly neophobic: n = 11 (12.6%) Neophilic: n = 10 (11.5%)

A post-hoc sensitivity analysis with *n* = 87 participants and 60 repetitions (stimuli per condition) at Bonferroni-corrected alpha of α = 0.00714 (see below) revealed an effect size of *d* = 0.32 at a power of 1- *β* = 0.8, and a power of 1- *β* = 0.53 for the initial effect size of *d* = 0.25.

### Stimulus material

Stimuli represent pairs of AI-generated and real matches of food and were validated in a pilot study. As the goal of the study is to compare real visual food stimuli to AI-generated counterparts with AI-generated stimuli as alternatives in the context of application contexts that would not contain generation artifacts, curation procedures were employed for the selection of AI-generated stimuli.

*Real stimuli.* For real food stimuli, an initial set of 96 real visual food stimuli were selected from the stimulus set used by Pimpini et al.^39^ and Kochs et al.^40^. Stimuli of those datasets include food stimuli selected from the Internet (e.g., Google, iStockphoto) and from prior food stimulus databases (food-pics_extended)^7,8^. The dataset stimuli were pre-formatted and pre-standardized with Adobe Photoshop CS5.1 and Matlab in terms of resolution (96 pixels/inch) and gray background color (RGB: 191, 191, 191). This stimulus set was used because its visual food stimuli are equally matched based on four categories based on caloric level (low/high) and flavor (sweet/savory), which provides a wide, balanced range of visual food stimuli.

*AI-generated stimuli*. AI-generated visual food stimuli were generated via Gemini 2.5 Flash (spring 2025) as paired counterparts to real images, leading to an initial total stimulus set of 192 stimuli. Stimulus generation followed a fixed prompt structure (Table 2). Because raw text-to-image output frequently contains artifacts and noise^41,42^, stimuli were generated iteratively and screened in two stages: (1) retaining the first output free of generation errors, and (2) validating recognizability and detectability in a pilot study. Curation of AI-generated stimuli occurred in line with practices for constructing AI stimulus sets for the selection of more realistic, artifact-free outputs^19,20,43^, especially when AI stimuli are generated to be used as research stimuli^43^.

Complete prompts that were used to generate the stimuli for each image are available at osf.io/qazu6. For each image, prompts were generated iteratively, and the first output free of generation errors was retained. Generation errors were a-priori defined as structural artifacts (e.g., incomplete or partial food, duplicated or merged items, distorted structure, generation noise). Food stimuli were selected based on the absence of artifacts and independent of the dimensions rated in the main experiment (realism, willingness to eat, healthiness, calorie content). When an image contained artifacts, the prompt was resubmitted with minimal re-generation instructions (e.g., “try again”). The number of generations required per dish was not systematically recorded. Post-processing was limited to matching AI-generated visual food stimuli and real counterparts in resolution and background color (96 pixels/inch, background RGB 191, 191, 191).

**Table 2:** General prompt structure as a template for the prompts used for the generation of each AI-generated visual food stimulus. Each stimulus prompt contained at least one of each prompt descriptor.

Prompt descriptor	Prompt content
Image generation command prompt	e.g., “generate an image using the following positive prompts”
Primary food descriptor	e.g., “realistic picture of almonds”, “create a round cake with powdered sugar (tiny particles) on top”, “create some rice waffles”
Secondary food descriptor	e.g., “without a plate”, “full view”
Background descriptor	“texture-less plain monotone grey background”
Realism/authenticity descriptors	e.g., “lifelike”, “natural”, “no shadow”, “not artistic”

*Pilot study*. To refine the food stimulus selection, an initial pilot study (*n* = 10) was conducted for which participants responded whether they (1) could recognize the specific dish, and (2) whether the visual food stimuli were real or AI-generated, in a two-alternative forced choice task, in order to select stimuli that were familiar and sufficiently realistic. Stimulus pairs with at least one stimulus below 66.67% recognizability and above 66.67% detection as AI-generated were removed. Number of pairs were then balanced by removing the amount of stimuli matching the category with the highest number of excluded stimuli (*n* = 9). Excluded stimuli and their reason of exclusion are summarized in Table A6.

*Final stimulus selection*. In total, 9 stimuli of each of the eight categories (2 calorie levels, 2 tastes, 2 stimulus type) were removed (72 in total; 36 real, 36 AI-generated), leading to a final selection of 120 stimuli (60 real, 60 AI-generated) with appropriate ratings of familiarity and realism. Selected visual food stimuli are summarized in Table 3. Example stimuli for each category are shown in Fig. 1.

**Table 3:** Visual food stimuli list (*n* = 60, 15 per category).

High-caloric savory	High-caloric sweet	Low-caloric savory	Low-caloric sweet
Avocado, Baguette, Brie, Fries, Ham, Herring, Hot dog, Olives, Pasta Pomodoro, Pizza, Ribs, Roasted potatoes, Salami, Smoked sausage, Sushi	Berliner, Cake, Candies, Pound cake, Lava cake, Chocolate ice-cream, Chocolate bar, Chocolate cake, Donut, Magnum, Mars, Nutella Sandwich, Tiramisu, Vanilla Ice-cream, Waffle	Asparagus green, Beetroot, Carrot, Cauliflower, Cherry tomatoes, Cod fish, Corn, Cucumber, Lettuce, Mushrooms, Oyster, Pop-corn, Radish, Rice waffle, Tuna	Apple, Banana, Blackberries, Cherries, Mandarine, Mango, Muesli bar, Papaya, Pear, Pomegranate, Raspberries, Red grapes, Strawberries, Watermelon, White grapes

### Questionnaires

To measure food evaluations, the scales *realistic*, estimated *healthiness*, estimated *calories*, and *willingness to eat* were used, according to previous research on the assessment of AI-generated and real visual food stimuli^15,17^. Each scale was presented with non-numerical subjective endpoint anchors to avoid anchoring effects via exact knowledge (e.g., for calorie content).

To measure food neophobia, a German translation of the Food Neophobia Scale was used^44^, consisting of 10 scales indicating how much participants agreed with statements such as “I am very selective about food”.

100-mm Visual Analogue Scales (VAS) were used for each assessment scale (realism, healthiness, calories, willingness to eat) with endpoints ranging from 0 (“not at all”) to 100 (“very much”). Food Neophobia scales were transformed into 100-mm VAS (0 = “strongly disagree”, 100 = “strongly agree”) to avoid potential anchoring effects of Likert scales, for internal consistency with the other scales, and to provide a parametric format more suitable for inferential statistics^45,46^. Likert-to-VAS transformations of personality questionnaires have been shown to yield comparable reliabilities, means, SDs, and criterion validity^24^.

### Procedure

The experiment was conducted online. After participants provided electronic informed consent, they filled out demographic and food neophobia questionnaires and were linked to a different website to conduct the experiment. During informed consent, participants were told that they would view multiple AI-generated and real images of food. To minimize information that may reveal the experimental condition, participants were not told that images were presented as matched real-AI counterparts, nor were they given information on whether stimuli were balanced. All visual food stimuli were presented in a randomized order and without source labels. For each scale, partifcipants had unlimited time to view and rate the image. Scales were presented in a fixed order (*realism*,* healthiness*,* calorie content*,* willingness to eat*) on ranges from 0 to 100.

### Data analysis

Data analysis was conducted using R (ver. 4.5.0). Outliers (defined as values more than 1.5 IQR spread from the median) were removed for each of the four scales according to preregistration (total: 975 values from 840 rows out of 9,700 rows (8.66%)). For real vs. AI-generated stimuli respectively, number of outliers were 139 vs. 67 (realism), 149 vs. 78 (willingness to eat), 103 vs. 114 (healthiness), and 162 vs. 163 (calorie content). Outliers were identified relative to the original dataset; hence, some trials (*n* = 135) contained outliers on multiple scales. Outlier removal had no effect on the significance of the main analysis results (see Table A1), indicating confidence in the results.

To minimize risk of bot contamination in data, any duplicate data detected via identical Prolific IDs were removed. In addition, any participant who took less than 16 min (half of the median: 31.53 min) was excluded from the study. Three such datasets were excluded from the study and replaced with three consecutively recruited participants, leading to a sample of *n* = 87.

Differences between real and AI-generated visual food stimuli were calculated as confirmatory analyses via linear mixed models with participant and food as random effects and random slopes and food condition (real vs. AI-generated) as fixed effect, predicting subjective ratings of realism, calorie content, healthiness, and willingness to eat. Alternative random effect structures were not compared. Linear mixed models were calculated using the package *lme4*^47^. Current hunger levels, food neophobia, and BMI were added as control variables.

Exploratory post-hoc analyses on the effects of stimulus properties (low/high calorie content, sweet/ savory flavor) were conducted by adding calorie content and flavor as additional fixed interaction effects to the LMMs simultaneously for full three-way interaction effects (condition × calorie content × flavor).

To assess whether the null condition effect of calorie ratings reflected general difficulty in calorie estimation, a post-hoc analysis was conducted to compare calorie-rating variability between conditions using a linear mixed model with participant as random effect and slope and condition-specific residual variances and compared it to a homoscedastic model by likelihood-ratio test. In addition, a linear mixed model was conducted to predict calorie ratings using normative calorie content (extracted for 51 of the 60 food dishes from the food-pics_extended database from which the real stimuli were derived^8^) and condition as fixed effects and stimulus and participant as random effects and slopes; a significant main effect of normative calorie content is interpreted as further validating the calorie rating scale.

To investigate AI hyperrealism, confirmatory analyses on differences in realism between conditions (main effect) and moderator analyses (moderator effect) were analyzed. Moderator analyses using linear mixed models with participant and food as random effects and random slopes and food-level differences in realism ratings (AI-generated minus real) as fixed effects were used to predict differences in calorie content, healthiness, and willingness to eat between AI-generated and real visual food stimuli. In addition, a post-hoc exploratory analysis was conducted to investigate whether realism ratings predict the other three ratings across stimulus conditions.

To investigate potential learning effects occurring throughout the experiment, an LMM on the interaction of condition and trial number (fixed effects) was conducted with food and participants as random effects and random slopes. A significant interaction effect of trial number is interpreted as a learning effect on realism ratings. To investigate direct carry-over effects between perceiving real and AI-generated counterparts, an LMM on the interaction of condition and counterpart proximity (difference of trial number between real and AI-generated stimuli) was conducted with food and participant as random effects and random slopes. Significant interaction effects between condition and proximity are interpreted as carry-over effects. Finally, an exploratory post-hoc analysis on sex effects on realism ratings was conducted with sex and condition as fixed factors and participant and food as random factors and random slopes.

Fixed effects were evaluated via non-directional omnibus tests. *β*-values are reported to interpret the directions of significant effects. As LMMs generate Satterthwaite approximations of degrees of freedom for each effect, degrees of freedom may differ between different effects of the same model.

To control for alpha inflation across seven confirmatory tests, a Bonferroni correction was applied to the alpha threshold of α = 0.05, leading to an adjusted significance threshold of α = 0.00714. To evaluate significance, *p*-values are compared to this adjusted significance threshold. Bonferroni corrections were applied to all confirmatory analyses.

Model assumptions were assessed graphically and not consistently met, and homoscedasticity of residuals was not met by any model. Heteroscedasticity may occur due to the bounded nature of VAS scales^48^ which may incline participants to use extreme values. Hence, robustness of results was confirmed using a logit transformation to stabilize variance, and a robust estimation of the models (via the *rlmer()* function from the package *robustlmm*^49^). Both analyses reproduced the main findings under the Bonferroni threshold (Table A5), indicating that the results are not artifacts of the response distribution.

## Data Availability

Data, analysis, and stimuli used in this study are publicly available at osf.io/qazu6.

## Appendix

**Table A1:** Comparisons of main result analyses between datasets with outliers removed and outliers not removed.

Analysis	Outlier removed	Outlier not removed
Realism	*F*(1,117) = 23.22, *p* < .001, *R*^2^_c_ = 0.50, β = 11.14	*F*(1,119) = 23.33, *p* < .001, *R*^2^_c_ = 0.48, β = 10.31
Willingness to eat	*F*(1,59) = 11.97, *p* = .001, *R*^2^_c_ = 0.30, β = 3.06	*F*(1,50) = 9.73, *p* = .003, *R*^2^_c_ = 0.27, β = 2.13
Calorie content	*F*(1,62) = 4.18, *p* = .045, *R*^2^_c_ = 0.83, β = −0.70	*F*(1,58) = 3.71, *p* = .059, *R*^2^_c_ = 0.77, β = −0.63
Healthiness	*F*(1,59) = 0.04, *p* = .834, *R*^2^_c_ = 0.86, β = −0.08	*F*(1,58) = 0.37, *p* = .543, *R*^2^_c_ = 0.83, β = −0.22

**Table A2:** Full test statistics including estimated marginal means (EMM), standard errors (SE), and 95% confidence intervals (CE) for each rating scale across conditions (real, AI-generated) and the difference between conditions.

Rating scale	Real: EMM (SE) [95% CI]	AI-generated: EMM (SE) [95% CI]	Difference: β (SE) [95% CI]	F(df1, df2)	*p*	*R* ^2^_c_
Realism	73.5 (1.95) [69.7 77.3]	62.4 (2.65) [57.2 67.5]	11.14 (2.31) [6.61 15.7]	*F*(1,117) = 23.22	< 0.001	0.50
Healthiness	51.6 (4.12) [43.6 59.7]	51.7 (4.06) [43.8 59.7]	-0.08 (0.38) [−0.83 0.67]	*F*(1,59) = 0.44	0.834	0.86
Calorie content	50.2 (3.81) [42.7 57.6]	50.9 (3.82) [43.4 58.4]	− 0.70 (0.34) [−1.38 −0.03]	*F*(1,62) = 4.18	0.045	0.83
Willingness to eat	67.8 (2.00) [63.9 71.8]	64.8 (1.91) [61.0 68.5]	3.06 (0.89) [1.35 4.81]	*F*(1,59) = 11.97	0.001	0.30

**Table A3:** Inferential statistics results of the effects of the control variables (Food Neophobia, BMI, acute hunger) on rating scales.

Rating scale	Food Neophobia	BMI	Acute hunger
Realism	*F*(1,82) = 0.01, *p* = .948	*F*(1,83) = 0.04, *p* = .850	*F*(1,80) = 0.94, *p* = .335
Healthiness	*F*(1,82) = 1.04, *p* = .311	*F*(1,82) = 0.01, *p* = .948	*F*(1,82) = 0.005, *p* = .948
Calorie content	*F*(1,82) = 0.01, *p* = .972	*F*(1,81) = 0.83, *p* = .364	*F*(1,81) = 0.35, *p* = .558
Willingness to eat	*F*(1,83) = 4.68, *p* = .033	*F*(1,85) = 0.30, *p* = .588	*F*(1,81) = 0.36, *p* = .552

**Table A4:** Inferential statistics (linear mixed models and contrasts with 95% confidence intervals on condition differences) results of the effects of sex and condition on realism ratings.

Effect	Statistics
Main effect of condition	*F*(1,113) = 0.08, *p* = .782
Main effect of sex	*F*(1,83) = 8.08, *p* = .006
Sex-condition interaction	*F*(1,83) = 11.64, *p* = .001
Female: condition difference (real – AI-generated)	*t*(131) = 3.18, *p* = .002 Difference = 7.69, CI [2.89, 12.5]
Male: condition difference (real – AI-generated)	*t*(131) = 6.06, *p* < .001 Difference = 18.66, CI [12.57, 24.8]

**Table A5:** Inferential statistics of the main analyses after variance-stabilizing logit transformation of the VAS data and robust estimations of the main analysis models. Robust estimation models do not define degrees of freedom.

Scale	Results: logit-transformed	Results: robust estimation
Realism	*F*(1,125) = 22.36, *p* < .001, *R*^2^_cor_ = 0.55	*t* = 21.42, *p* < .001
Calories	*F*(1,60) = 5.58, *p* = .021, *R*^2^_cor_ = 0.76	*t* = −2.11, *p* = .035
Healthiness	*F*(1,60) = 0.16, *p* = .695, *R*^2^_cor_ = 0.78	*t* = 0.13, *p* = .893
Willingness to eat	*F*(1,60) = 12.40, *p* < .001, *R*^2^_cor_ = 0.31	*t* = 4.76, *p* < .001

**Table A6:** Food items removed in the pilot study, by food type, and reason of exclusion. *AI det. > 66%* means the AI-generated stimulus was detected as AI-generated more than 66% of the time. *Rec. < 66.67%* means the stimulus (real or AI-generated) was recognized less than 66.67% of the time. *Balancing* means the stimulus was removed to balance the number of stimuli across categories.

High caloric savory	High caloric sweet	Low caloric savory	Low caloric sweet
Almonds (AI det. > 66.67%)	Bonbon (AI det. > 66.67%)	Aubergine (balancing)	Apricot (AI det. > 66.67%)
Bacon (AI det. > 66.67%)	Brownies (AI det. > 66.67%)	Broccoli (balancing)	Coconut (AI det. > 66.67%)
Bitterballs (rec. < 66.67%)	Butter biscuits (rec. < 66.67%)	Brussel sprouts (AI det. > 66.67%)	Currants (AI det. > 66.67%)
Blue cheese (AI det. > 66.67%)	Cheese cake (AI det. > 66.67%)	Chickpeas (AI det. > 66.67%)	Figs (AI det. > 66.67%)
Cheese soufflé (rec. < 66.67%)	Choco cookies (AI det. > 66.67%)	Mussel (rec. < 66.67%)	Honey melon (AI det. > 66.67%)
Chips (AI det. > 66%)	Cream cake (AI det. > 66%)	Peas (AI det. > 66%)	Kiwi (AI det. > 66.67%)
Fried eggs (AI det. > 66.67%)	Croissant (AI det. > 66.67%)	Red cabbage (AI det. > 66.67%)	Melon (AI det. > 66.67%)
Hamburger (AI det. > 66.67%)	M&Ms (AI det. > 66.67%)	Red pepper (AI det. > 66.67%)	Orange (AI det. > 66.67%)
Lasagne (AI det. > 66.67%)	Tompouce (AI detected > 66.67%)	Zucchini (AI det. > 66.67%)	Pineapple (balancing)
